# Posterior Semicircular Canal Dehiscence with Vestibulo-Ocular Reflex Reduction for the Affected Canal at the Video-Head Impulse Test: Considerations to Pathomechanisms

**DOI:** 10.3390/audiolres14020028

**Published:** 2024-03-24

**Authors:** Andrea Castellucci, Georges Dumas, Sawsan M. Abuzaid, Enrico Armato, Salvatore Martellucci, Pasquale Malara, Mohamad Alfarghal, Rosanna Rita Ruberto, Pasquale Brizzi, Angelo Ghidini, Francesco Comacchio, Sébastien Schmerber

**Affiliations:** 1 ENT Unit, Department of Surgery, Azienda USL—IRCCS di Reggio Emilia, 42123 Reggio Emilia, Italy; angelo.ghidini@ausl.re.it; 2 EA 3450 DevAH–Development, Adaptation and Handicap, Faculty of Medicine, University of Lorraine, 54500 Nancy, France; georges.dumas10@outlook.fr; 3 Otorhinolaryngology Department, Royal Medical Services, Amman 11855, Jordan; s.abuzaid81@yahoo.com; 4 Ph.D. Program in Development, Adaptation and Handicap, Faculty of Medicine, University of Lorraine, 54500 Vandoeuvre-lès-Nancy, France; armato.otovest@gmail.com; 5 ENT Unit, Santa Maria Goretti Hospital, Azienda USL di Latina, 04100 Latina, Italy; dott.martellucci@gmail.com; 6 Audiology & Vestibology Service, Centromedico, 6500 Bellinzona, Switzerland; pasmalara@gmail.com; 7 Otorhinolaryngology—Head and Neck Section, Surgery Department, King Abdulaziz Medical City, Jeddah 21556, Saudi Arabia; audio1972@gmail.com; 8 Audiology and Ear Surgery Unit, Azienda USL—IRCCS di Reggio Emilia, 42123 Reggio Emilia, Italy; rosannarita.ruberto@ausl.re.it (R.R.R.); pasquale.brizzi@ausl.re.it (P.B.); 9 ENT Unit, Regional Vertigo Specialized Center, University Hospital of Padova, Sant’Antonio Hospital, 35039 Padova, Italy; comacchio.francesco@alice.it; 10 Department of Oto-Rhino-Laryngology, Head and Neck Surgery, University Hospital, 38043 Grenoble, France; sschmerber@chu-grenoble.fr

**Keywords:** posterior canal dehiscence, third mobile window, horizontal canal dehiscence, video-head impulse test, vestibular evoked myogenic potentials, conductive hearing loss, Meniere’s disease

## Abstract

Posterior semicircular canal dehiscence (PSCD) has been demonstrated to result in a third mobile window mechanism (TMWM) in the inner ear similar to superior semicircular canal dehiscence (SSCD). Typical clinical and instrumental features of TMWM, including low-frequency conductive hearing loss (CHL), autophony, pulsatile tinnitus, sound/pressure-induced vertigo and enhanced vestibular-evoked myogenic potentials, have been widely described in cases with PSCD. Nevertheless, video-head impulse test (vHIT) results have been poorly investigated. Here, we present six patients with PSCD presenting with a clinical scenario consistent with a TMWM and an impaired vestibulo-ocular reflex (VOR) for the affected canal on vHIT. In two cases, an additional dehiscence between the facial nerve and the horizontal semicircular canal (HSC) was detected, leading to a concurrent VOR impairment for the HSC. While in SSCD, a VOR gain reduction could be ascribed to a spontaneous “auto-plugging” process due to a dural prolapse into the canal, the same pathomechanism is difficult to conceive in PSCD due to a different anatomical position, making a dural herniation less likely. Alternative putative pathomechanisms are discussed, including an endolymphatic flow dissipation during head impulses as already hypothesized in SSCD. The association of symptoms/signs consistent with TMWM and a reduced VOR gain for the posterior canal might address the diagnosis toward PSCD.

## 1. Introduction

Semicircular canal dehiscence (SCD) represents a pathological condition of the inner ear, which has gained attention during the last 25 years thanks to the description of superior SCD (SSCD) syndrome [1]. It represents the archetype of third-window pathologies, where a bony canal defect generates a low-impedance pathway for air-conducted (AC) sounds and pressure stimuli leading to an array of peculiar symptoms and signs. The pathomechanism underlying SSCD is also called the third-mobile window mechanism (TMWM), as the distinctive low-frequency air-bone gap (ABG) on audiometry with normal tympanometry, the characteristic nystagmus aligning with the affected SC in response to sound/pressure stimuli and skull vibrations and the enhanced vestibular-evoked myogenic potentials (VEMPs) have been attributable to the introduction of an additional window in the vestibular partition of the inner ear. Therefore, the typical clinical picture consistent with a TMWM includes vestibular symptoms such as sound/pressure-induced nystagmus (also known as Tullio phenomenon and Hennebert’s sign, respectively) and chronic imbalance, and auditory manifestations such as autophony, aural fullness, bone-conducted (BC) sound hyperacusis, hearing loss (HL) and pulsatile tinnitus [1,2,3,4,5,6,7,8]. Even though aural fullness and autophony can also be found in both patulous Eustachian tube and SCD, patients with a patulous Eustachian tube generally have autophony for their own breath sounds, while patients with SCD usually do not [9]. Besides SCD, other conditions resulting in a TMWM have been described over the years, including an enlarged vestibular aqueduct, X-linked gusher disorder and cochlear dehiscence either involving the internal carotid artery or the facial nerve (FN) [3,5,6,10,11,12,13,14]. Despite being rare, posterior SCD (PSCD) represents the second most common SCD after SSCD, with a 2% incidence among adult patients and a variable incidence ranging from 1.3 to 43% in the pediatric population [15,16,17,18]. This huge variability likely depends on several factors, including the type of surveys (histopathological versus radiological), the age of the cohort and the adopted slice thickness for CT scan. Although it is usually associated with a high-riding jugular bulb (HJB), cases with PSCD at the posterior cranial fossa (PCF) have been reported, even in association with other SCD [13,15,18,19,20,21,22,23]. According to a recent proposal for a unitary classification of all otic capsule dehiscences, PSCD due to a HJB should be classified as vascular extralabyrinthine dehiscence (type II), while PSCD at the PCF should be classified as meningeal dehiscence (type I) [11]. Furthermore, even though PSCD has been demonstrated to result in a clinical and instrumental phenotype overlapping SSCD, cases presenting with symptoms and signs inconsistent with a TMWM, including atypical Meniere’s disease (MD), have been also described [4,18,24,25,26]. Possible differential diagnosis includes ossicular discontinuity, malleus head fixation, Paget’s disease, osteogenesis imperfecta and otosclerosis [3,27].

A high-resolution CT (HRCT) scan of temporal bones with parasagittal reconstructed images along the PSC plane (Stenver plane) represents the gold standard for diagnosis [2,24]. Conversely, horizontal SCD (HSCD) is generally caused by an erosive process of the middle ear, which usually masks symptoms and signs due to a TMWM [4,28,29]. Nevertheless, HSCD has been anecdotally detected in cases of normal middle ear status [30,31]. Recently, the dehiscence between the HSC and the tympanic tract of the FN has been described as a potential cause of a TMWM [12,13]. In this case, reformatted images along the SSC plane (Pöschl plane) can better visualize the bony defect.

The video-head impulse test (vHIT) has recently become a feasible tool to assess the vestibulo-ocular reflex (VOR) gain of the six semicircular canals (SCs) in the high-frequency domain. Different devices with either a head-mounted or remote camera and with different algorithms for the VOR gain calculation have been demonstrated to yield roughly similar and repeatable results [32,33]. Thanks to its easy accessibility, in recent years, the vHIT has become the key office procedure to test SC activity in both acute and chronic vestibular disorders, allowing clinicians to detect peculiar lesion patterns depending on the underlying pathomechanisms and etiologies by matching vHIT data with audiometric and VEMPs results [34,35,36,37,38,39,40,41]. Nevertheless, few studies have investigated vHIT data in SCD so far [42,43,44,45,46,47,48]. Most of them have focused on the modification of the VOR gain of the affected SC in SSCD, where a hypothetical spontaneous prolapse of the middle cranial fossa into the canal through the bony dehiscence is assumed, resulting in a natural occlusion of the membranous duct and in an impaired SSC activity. Recently, alternative mechanisms have been offered to explain how some SSCD patients with SSC hypofunction on vHIT still exhibit symptoms and signs consistent with a TMWM. In fact, a TMWM is expected to fade after a spontaneous canal occlusion, similarly to what it is expected from a surgical canal plugging [48,49,50,51]. An incomplete canal occlusion or a membranous canal indentation at the dehiscence leading to an endolymphatic flow dissipation during impulsive tests have been recently hypothesized [47,49]. On the other hand, VOR gain data have been only anecdotally reported in cases of PSCD, yielding conflicting results [18,42,43], while there are no data of vHIT measurements in idiopathic HSCD. Here, we describe six patients with PSCD presenting with symptoms and signs consistent with a TMWM and with reduced VOR gain values for the involved PSC on vHIT. An additional dehiscence between the ampullary arm of the HSC and the intra-tympanic tract of the FN was detected in two cases, leading to a concurrent VOR impairment for the HSC. We aim to provide possible explanations for these findings.

## 2. Case Description

### 2.1. Case 1

**Figure 1:**
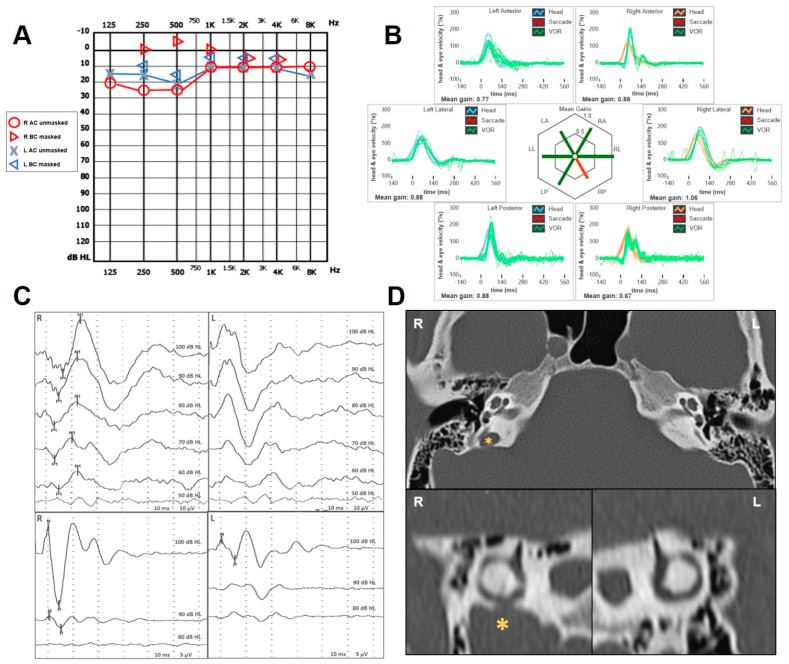
Instrumental findings of case 1. (**A**) Pure-tone audiometry showing low-frequency pseudo-CHL on the right side and normal hearing threshold on the left. (**B**) vHIT data using the ICS Impulse device (GN Otometrics, Taastrup, Denmark) with a head-mounted camera, assessing all six semicircular canal VOR in horizontal, LARP, and RALP planes. Mean value of VOR gain (eye velocity/head velocity) is reported for each canal. Gains are considered normal if >0.8 for lateral canals and >0.7 for vertical canals. A selective slight reduction in the VOR gain for the right PSC (0.65) with covert saccades was registered. (**C**) Cervical (above) and ocular VEMPs (below) for AC sounds were recorded using a two-channel evoked potential acquisition system (Viking, Nicolet EDX, CareFusion, Heidelberg, Germany). Potentials were recorded delivering tone bursts (frequency: 500 Hz, duration: 8 ms, stimulation rate 5 Hz) via headphones. VEMP testing revealed low thresholds (60 dB nHL) on the right side for cervical VEMPs and enlarged right-sided amplitudes (R: 69 μV and L: 17 μV for 100 dB nHL stimuli) for ocular VEMPs. (**D**) Axial images (above) of temporal bone HRCT scan with parasagittal reconstructions along the Stenver planes (below), showing a dehiscence between the right PSC and a HJB (yellow asterisk).

An 18-year-old female presented with progressive-onset pulse-synchronous tinnitus in her right ear, which increased during physical efforts and in supine position. She also described ipsilateral aural fullness, autophony and own-body sound hyperacusis. Neither head trauma nor chronic pathology was reported. Otoscopy was normal. Pure-tone audiometry showed a right-sided pseudo-conductive HL (CHL) for the low frequencies with negative values of BC thresholds at 250 Hz and normal hearing on the left side (Figure 1A), while impedance audiometry revealed normal tympanometry and acoustic reflexes on both sided. Even though she denied vestibular symptoms, a complete vestibular assessment was administered. While Video-Frenzel examination was unremarkable, including a head-shaking test and Valsalva maneuvers, a slight VOR gain impairment (0.67) for the right PSC was detected at the vHIT (Figure 1B). Both cervical and ocular VEMPs to AC sounds revealed enhanced amplitudes and reduced thresholds on the right side (Figure 1C). The patient received a temporal bone HRCT scan that showed a PSCD due to a HJB on the right side (Figure 1D). The gadolinium-enhanced brain MRI was unremarkable. Though a surgical treatment either with a transmastoid PSC occlusion or an endovascular stenting of the HJB was proposed, she opted for a follow-up strategy as symptoms were tolerable.

### 2.2. Case 2

**Figure 2:**
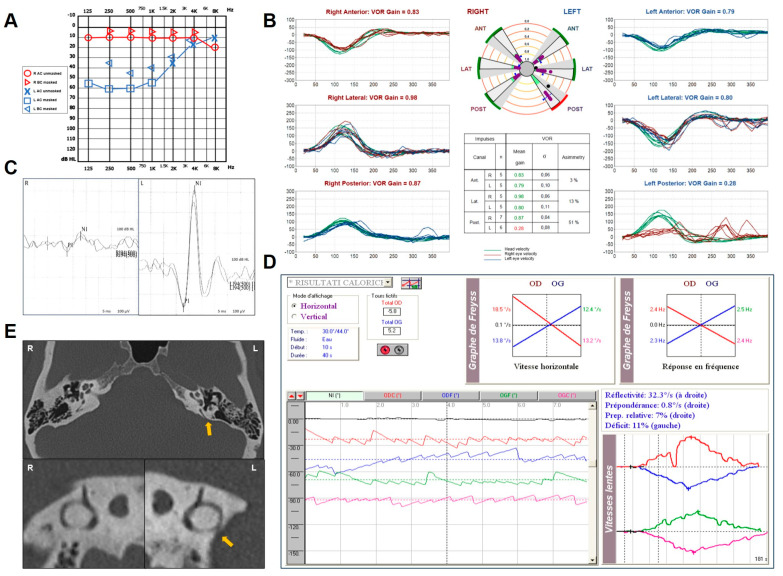
Instrumental findings of case 2. (**A**) Pure-tone audiometry showing normal hearing threshold on the right and up-sloping mixed HL on the left side with low-frequency ABG. (**B**) vHIT measurements using the Ulmer system (Synapsys, Marseille, France) with a remote camera. A selective VOR gain impairment for the left PSC (0.28) with overt saccades was registered. (**C**) Cervical VEMPs for AC sounds recorded using the ICS Chartr EP 200 system (GN Otometrics, Taastrup, Denmark) revealing enhanced amplitudes on the left side (R: 82 μV and L: 490 μV for 100 dB nHL stimuli). (**D**) BCT performed with a VNG device (Synapsys, Marseille, France) showing symmetrical responses (**E**). Temporal bone HRCT scan with axial (above) and parasagittal images (below) along the Stenver planes showing, on the left, a PSCD at the PCF (yellow arrow).

A 44-year-old female with chronic autoimmune thyroiditis was re-evaluated by our team for a 6-year history of fluctuating auditory symptoms, including low-frequency sensorineural HL (SNHL), aural fullness and pulsatile tinnitus. She also reported slight unsteadiness and occasional acute vertigo spells. She was already diagnosed with a left-sided MD and submitted to uneventful dietary restrictions, oral steroid treatment, e.v. glycerol administration and intratympanic injections of 4 mg/mL Dexamethasone. Nevertheless, her left hearing function worsened and she started to develop recurrent benign paroxysmal positional vertigo (BPPV) involving the left PSC. A gadolinium-enhanced brain MRI was negative. The patient was submitted to a complete audio-vestibular assessment. A pure-tone audiogram depicted a normal hearing function on the right and a mixed HL with an up-sloping sensorineural component and low-frequency ABG on the left side (Figure 2A). Impedance audiometry was normal. The video Frenzel examination showed a slight right-beating nystagmus after head shakings, while the vHIT showed a significant reduction (0.28) in the left PSC VOR gain (Figure 2B). Abnormally enhanced cervical VEMPs to AC stimuli were detected on the left side, while normal HSC reflectivity in the low-frequency domain was measured on bithermal caloric test (Figure 2C,D). A temporal bone HRCT highlighted a left-sided PSCD dehiscence at the PCF (Figure 2E). Due to the persistence of disabling symptoms, the patient was submitted to a transmastoid PSC plugging with bone dust, temporalis fascia and bone pâté. After surgery, she transiently experienced severe imbalance due to the onset of an acute global left vestibular hypofunction, with no hearing impairment. At 1 month, left HSC and SSC function recovered, while the PSC activity remained impaired. At 1-year follow-up, the patient fully recovered from vestibular symptoms, while tinnitus and aural fullness progressively improved. Low-frequency ABG disappeared.

### 2.3. Case 3

**Figure 3:**
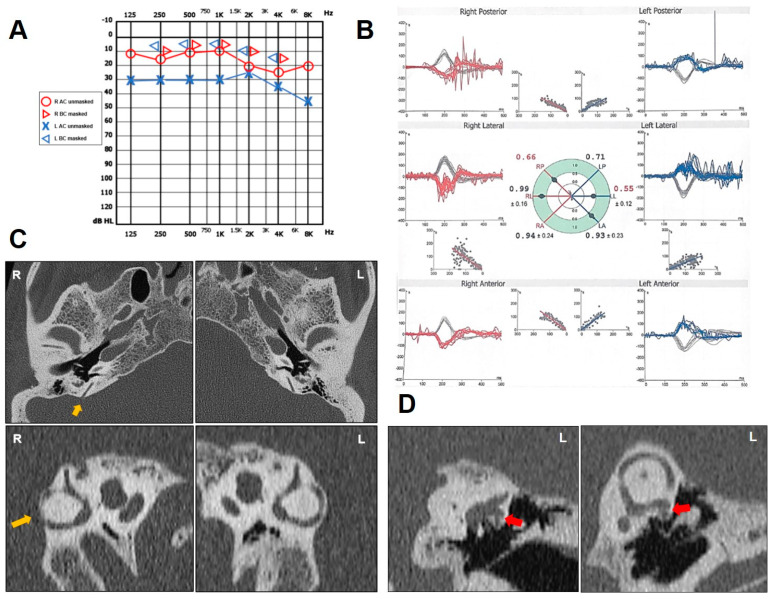
Instrumental findings of case 3. (**A**) Pure-tone audiometry showing normal hearing threshold on the right side and flat CHL on the left. (**B**) vHIT data using a device with head-mounted camera (Difra, Eupen, Belgium). Right PSC and left HSC exhibited reduced VOR gain values (0.66 and 0.55, respectively) with both overt and covert saccades. (**C**) Axial images (above) of temporal bone HRCT scan with parasagittal reconstructions along the Stenver planes (below), showing a dehiscence between the right PSC and the PCF (yellow arrow). (**D**) Coronal slice (left panel) and parasagittal reconstruction along the Pöschl plane of the left ear (right panel) highlighting a small dehiscence between the ampullary arm of the HSC and the FN (red arrow).

A 59-year-old man with a history of chronic imbalance for 3 years was evaluated for the progressive onset of bilateral autophony and pulsatile tinnitus. He also reported a long-lasting history of left-sided HL and recurrent ear discharge. A dry central perforation of the left TM was detected on otoscopy. Pure-tone audiometry showed a normal hearing threshold on the right side and a slight flat CHL on the left (Figure 3A). The video Frenzel examination was overall unremarkable. Conversely, a slight VOR gain reduction for the right PSC and the contralateral HSC was detected on vHIT (Figure 3B). AC cervical VEMPs showed low thresholds on the right (60 db nHL) consistent with a TMWM, and ocular VEMPs were confirmed as repeatable in response to 4 kHz tone bursts ipsilaterally. The presence of the left TM perforation precluded an adequate interpretation of the VEMPs on the left side, as BC stimulation was not available in our institute. A temporal bone HRCT scan showed a clear right-sided PSCD at the PCF (Figure 3C). Since autophony and tinnitus were bothersome on the right side, a round window (RW) reinforcement was pursued in general anesthesia on the right side, with partial improvement in symptoms. After 8 months, the patient reported an increasing of auditory symptoms on the left. Brain MRI was unremarkable. The HRCT scans were reviewed and a tiny dehiscence between the ampullary arm of the left HSC and the FN was highlighted (Figure 3D). He was then scheduled for a tympanoplasty on the left side with simultaneous RW reinforcement.

### 2.4. Case 4

**Figure 4:**
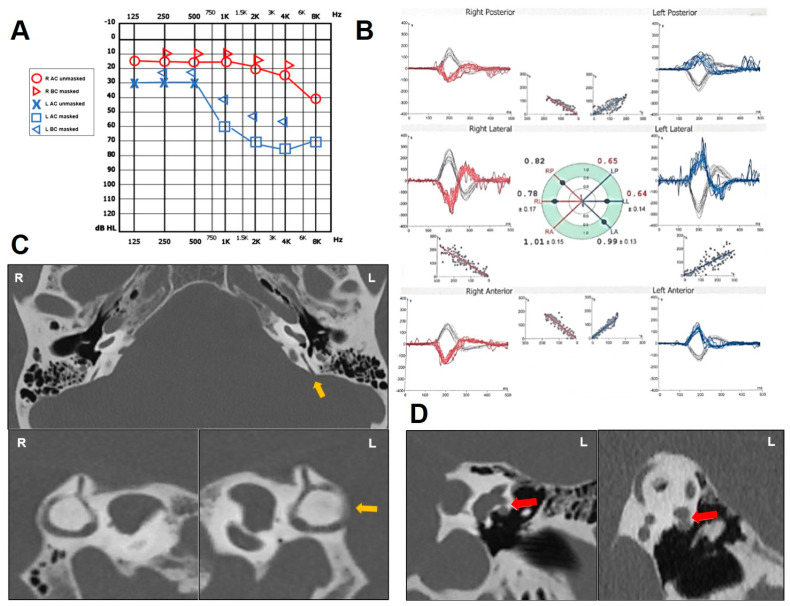
Instrumental findings of case 4. (**A**) Pure-tone audiometry showing normal hearing threshold on the right side and down-sloping SNHL with mild/high-frequency ABG on the left. (**B**) vHIT data (Difra, Eupen, Belgium) exhibiting slightly reduced VOR gain values for both HSC and PSC on the left side (0.64 and 0.65, respectively) with mainly covert saccades. (**C**) Axial images (above) of temporal bone HRCT scan with parasagittal reconstructions along the Stenver planes (below), showing a dehiscence between the left PSC and the PCF (yellow arrow). (**D**) Coronal slice (left panel) and parasagittal reconstruction along the Pöschl plane of the left ear (right panel) highlighting a small dehiscence between the ampullary arm of the HSC and the FN (red arrow).

A 68-year-old man with a history of HL, pulsatile tinnitus and autophony on his left ear developed positional vertigo spells. In particular, he experienced unsteadiness when lying supine from the sitting position. Neither head trauma nor chronic pathology except for arterial hypertension were reported. Otoscopy was unremarkable on both sides. Pure-tone audiometry showed a slight SNHL for high tones on the right side and a down-sloping SNHL with a high-frequency ABG on the left side (Figure 4A). Tympanometry and acoustic reflexes were bilaterally normal. A standard brain MRI excluded retrocochlear pathologies. Despite video Frenzel examination being overall negative, cervical VEMPs were enlarged with low thresholds (70 db nHL) on the left side and the vHIT highlighted a slight VOR gain reduction for both the PSC and the HSC on the left side (Figure 4B). A temporal bone HRCT scan depicted a PSCD at the PCF (Figure 4C) and a dehiscence between the ampullary arm of the left HSC and the FN (Figure 4D). Since his symptoms were not incapacitating, he was only suggested to avoid loud sounds, strains, and prolonged positionings with deep head hanging.

### 2.5. Case 5

**Figure 5:**
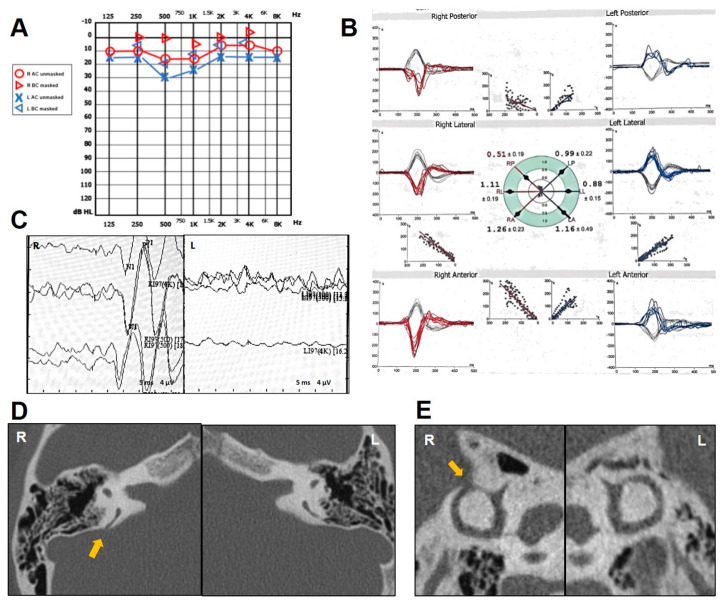
Instrumental findings of case 5. (**A**) Pure-tone audiometry with mild-low-frequency ABG on the right and normal hearing on the left. (**B**) vHIT measurements (Difra, Eupen, Belgium) exhibiting a selective VOR gain impairment for the right PSC (0.51) with mainly covert saccades. (**C**) Ocular VEMPs for AC sounds (ICS Chartr EP 200 system, GN Otometrics, Taastrup, Denmark) revealing very large amplitudes on the right side (15 μV) in response to 500 Hz stimuli and clearly repeatable waves at 4 kHz stimuli. Temporal bone HRCT scan with axial (**D**) and parasagittal images (**E**) showing a PSCD at the PCF on the right (yellow arrow).

A 13-year-old girl presented with recurrent spells of positional vertigo for 2 years. She also complained of sound-induced unsteadiness, dizziness when blowing her nose and subtle pulsatile tinnitus in her right ear. Neither head trauma nor inner ear pathology were reported by her parents. While normal TM were detected on otoscopy, audiometry showed a slight CHL for the mild-low frequencies on the right side with normal impedance audiometry (Figure 5A). Video-Frenzel examination was unremarkable at the time of the evaluation. While a selective reduced VOR-gain value for the right PSC was detected on vHIT (Figure 5B), right ocular VEMPs showed large amplitudes and clearly repeatable waves at 4 kHz stimuli (Figure 5C). Temporal bone HRCT scan highlighted a PSCD at the PCF on the right side (Figure 5D,E). Due to the young age of the patient and the mild symptoms, both the patient and her parents were suggested to avoid triggers and to keep a strict clinical follow-up.

### 2.6. Case 6

**Figure 6:**
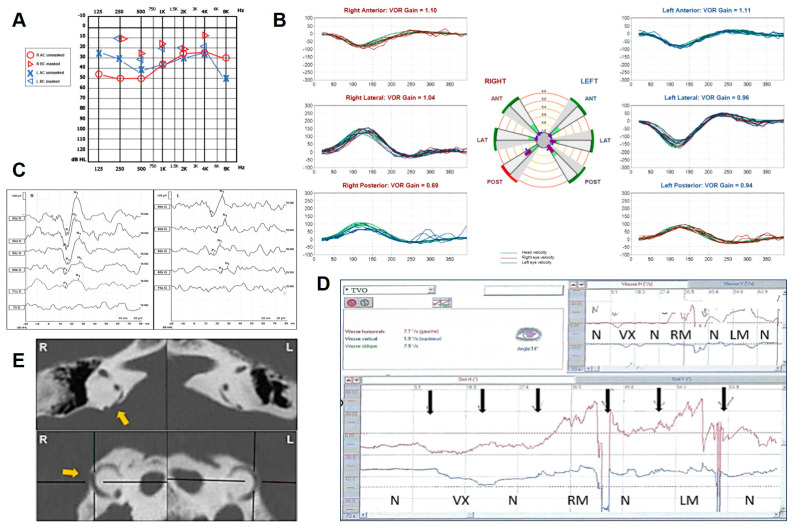
Instrumental findings of case 6. (**A**) Pure-tone audiometry showing bilateral mixed HL with mild/low-frequency ABG on the right side. (**B**) vHIT measurements (Synapsys, Marseille, France) with a slight selective VOR gain impairment for the right PSC (0.67) with overt saccades. (**C**) Cervical VEMPs for AC sounds showing enhanced amplitudes (R: 103.8 μV and L: 58.2 μV for 95 dB nHL stimuli) and lowered thresholds (R: 75 dB nHL, L: 85 dB nHL) on the right side. (**D**) Skull vibration induced nystagmus results: Video-nystagmographic recording (Synapsys, Marseille, France) showing leftbeating nystagmus (red lines) with slight upbeat components (black lines) during right and left mastoid stimulations (RM and LM, respectively). No response observed during vertex (VX) stimulation in this order of stimulation (N = no stimu-lation, Vx = vertex stimulation, RM = right mastoid stimulation, LM = left mastoid stimulation). (**E**) Axial (above) and reformatted images along the Stenver plane of the temporal bone HRCT showing on the right a PSCD at the PCF (yellow arrow) and an extreme thinning of the contralateral PSC.

A 65-year-old woman with progressive bilateral HL presented with unsteadiness for 3 years and recurrent positional vertigo spells with no signs of BPPV. She also complained of intolerance to loud sounds, right aural fullness with no autophony. Her clinical history was consistent with chronic bronchitis, asthma, diabetes and moderate obstructive sleep apneas treated with a nocturnal positive-pressure ventilation. Normal TM was found on otoscopy, while audiometry showed a mixed HL on both sides, with predominant CHL for the mild-low frequencies on the right side (Figure 6A). vHIT testing resulted in a selective slight hypofunction of the right PSC (Figure 6B) and right cervical VEMPs showed enlarged and low-threshold potentials (Figure 6C). While the Hennebert sign was negative, a left-beating nystagmus with slight upbeating components was recorded on video-oculography while stimulating her mastoids with a 100 Hz vibrator; conversely, no nystagmus was elicited while vibrating on the vertex (Figure 6D). Even though the temporal bone HRCT scan showed a PSCD at the PCF on the right side and an extreme thinning of the left PSC (Figure 6E), the patient refused surgical therapy, so she was suggested to avoid triggers and continue with a clinical follow-up.

Written informed consent was obtained from all the patients to publish this case report, including all data and images.

## 3. Discussion

According to the literature review, several peripheral vestibular disorders can lead to a selective SC impairment in the vHIT through different pathomechanisms. Theoretically, selective lesions involving either the HSC or the SSC are less likely given that both SCs share the same innervation through the superior vestibular nerve and the same vascular supply through the anterior vestibular artery. Therefore, an isolated HSC involvement on vHIT has been most frequently depicted in MD [52,53], while it has been rarely described in vestibular neuritis [54,55] and only anecdotally in cases of canalith jam [37]. On the other hand, an isolated SSC hypofunction has been mostly described in SSCD [44,45,46,49,50] and only sporadically in some variants of SSC-BPPV [39]. Conversely, the PSC is more frequently involved SC alone, as both acute and chronic diseases affecting vestibular afferents, such as vestibular neuritis and vestibular schwannoma, respectively, can lead to an isolated SC impairment only when involving the inferior vestibular nerve [34,35,36,40,56]. Similarly, acute ischemic lesions of the labyrinth can result in a selective SC dysfunction only when the common cochlear artery or its branches are involved, resulting in PSC and saccular involvement in association with sudden SNHL [36,40,41]. Besides the aforementioned dysfunctions, other inner ear disorders can account for a selective PSC impairment at the vHIT, including MD either in the acute or inter-ictal stage and atypical variants of PSC-BPPV [36,39,40,53,57,58,59]. In these cases, alterations of the intralabyrinthine micromechanics should be considered as the underlying pathomechanism, likely because the physiological undermost position of the PSC increases its vulnerability to acute/chronic damages on a hydropic basis and to a sudden blockage of the endolymphatic flow due to otoconial dislodgements [38,39,40,58,59]. In the cases with PSCD herein reported, other pathomechanisms likely accounted for the selective PSC hypofunction. Unlike SSCD exhibiting a middle fossa dura overlying the canal defect, a spontaneous PSC plugging exerted by the PCF dura seems hardly conceivable. First, a natural PSC occlusion should have resulted in an abolition or mitigation of symptoms and signs consistent with a TMWM, contrary to what has been found in our patients. In fact, an ABG closure and VEMP normalization, besides a mitigation/resolution of SCD symptoms, should have theoretically been expected after a canal occlusion, either natural or surgical [1,6,50,51,60]. On the contrary, all our patients presented with a clinical/instrumental picture consistent with a TMWM, as if the PSCD were particularly patent, fulfilling diagnostic criteria for SCD [61]. In particular, 83% of patients presented with pulsatile tinnitus, while 50% of cases reported HL, autophony, aural fullness, unsteadiness and/or vertigo spells. Sound/pressure unsteadiness was reported in two cases, while hyperacusis only in one case. One patient (case 2) presented with symptoms consistent with atypical MD. From an instrumental point of view, 83% of patients exhibited an ABG with normal tympanometry, including one case with a supranormal BC threshold consistent with a pseudo-CHL [2,3,26], while enhanced VEMPs could be detected in all cases, including repeatable ocular responses up to 4 kHz AC stimuli in two cases, which seems to represent a specific finding for SCD [62] (see Table 1). The skull vibration test was performed only in one case (case 6), resulting in a nystagmus with a slight up-beating component as expected from a PSC activation [63]. Actually, it could be also assumed that a herniation of the dura might account for a less effective canal occlusion compared to the surgical materials, resulting in an incomplete or intermittent canal plug and in an incomplete TMWM [45,47,50]. Nevertheless, the anatomical position of the PSC relative to the PCF, per se, makes a dural prolapse less likely, unless cerebrospinal fluid pressure increase occurs. According to these assumptions, this putative process might only occur in the case of PSCD due to a HJB (case 1), where the pulsating activity of the venous structure might be strong enough to overcome gravity and occlude the membranous duct of the PSC. As for case 2, given the history consistent with ipsilesional MD, it could also be hypothesized that a hydropic distension of the undermost part of the labyrinth (PSC and saccule) might lead to a selective PSC loss, either directly or indirectly, through a distension of the otolith structure herniating into the PSC [64]. This assumption could be supported by the detection of signs of endolymphatic hydrops in patients with TMW disorders [65,66,67]. Nevertheless, the instrumental profile of the patient was fully consistent with a TMWM and symptoms improved after PSC plugging, while standard treatment attempts for MD were ineffective, making the aforementioned mechanism less likely. Conversely, as already hypothesized for those SSCD cases with SSC hypofunction exhibiting a clinical/instrumental profile consistent with a TMWM, an endolymphatic flow dissipation at the dehiscence during head impulses leading to an “apparent” VOR gain impairment for the affected canal might represent an alternative explanation [47,49]. In other words, the loss of the bony canal integrity, that in normal condition should correctly drive the high-acceleration flow energy along the canal plane (i.e., away or toward the SC ampulla) during head impulses, might likely account for a membranous canal indentation at the dehiscence on a transient basis, leading to a dissipation of endolymphatic flow energy during head impulses. Therefore, the amount of the fluid mechanical wave running along the plane of the canal (which should efficiently deflect the PSC cupula to fully activate the canal afferents during upward head impulses) might be reduced, thus accounting for an “apparent” PSC VOR gain hypofunction (or VOR gain “pseudo-hypofunction”) at the vHIT [47,49]. Similar mechanisms involving membranous duct indentations have been demonstrated in experimental models of SSCD in response to loud sounds, pressure stimuli and skull vibrations [6,7,8]. Therefore, the same low-impedance pathway generating a clinical/instrumental profile consistent with a TMWM, such as nystagmus evoked by sound/pressure stimuli or skull vibrations, low frequency CHL and enhanced VEMPs, could also account for the reduced VOR-gain values for the dehiscent canal. Accordingly, PSCD should be included among the heterogeneous group of inner ear disorders accounting for a selective PSC VOR-gain reduction [36,40]. Similarly, the aforementioned hypothetical mechanisms could be assumed to explain the concurrent HSC hypofunction in both patients with radiological evidence of HSC-FN dehiscence (cases 3 and 4). In fact, the lack of bony coverage at the ampullary arm of the HSC might result in an endolymphatic flow dissipation at the dehiscence during horizontal head impulses, thus reducing the HSC VOR-gain. Even in these cases, the anatomical disposition of the FN with respect to the HSC makes a spontaneous HSC plug rather unlikely. Therefore, as already theorized for SSCD, an “apparent” SC hypofunction could represent an additional sign of TMWM and SCD should be always suspected if patients with a clinical/instrumental profile recalling a TMWM exhibit a selective SC impairment at the vHIT [49].

Nevertheless, it should be reminded that the patients herein described only represent a part of our cohort of PSCD (approximately 60%, unpublished data) which includes subjects exhibiting normal VOR-gain measurements for the affected PSC. Therefore, similar to SSCD, additional factors such as dehiscence size and location, membranous canal impedance and different degrees of compliance of the surrounding structures might account for the wide range of symptoms and instrumental data [44,68,69]. The same factors might also account for the heterogeneous clinical profile detected in our patients. Moreover, it was not possible to visualize a potential spontaneous natural plugging of the affected SC in these patients, as 3D-inner ear MRI sequences were not included in our working methodology. Future studies involving larger cohorts and including dedicated high-quality 3D-imaging are needed to strengthen the validity of our considerations.

Finally, two patients developed disabling symptoms related to a TMWM and were operated on either through canal plugging via a transmastoid approach (case 2) or through a RW reinforcement (case 3). While the first technique has been demonstrated to represent an effective treatment option for third window symptoms, RW reinforcement represents a viable choice for some patients [10,70]. Accordingly, both patients reported an improvement in both auditory and vestibular symptoms after surgery. The lady who underwent canal plugging developed a transient global vestibular hypofunction overlapping the post-operative findings in patients after SSC occlusion in SSCD [48,50,51]. On the other hand, the man who underwent RW reinforcement developed symptoms due to a contralateral HSCD with the FN after surgery, similar to what has been described in patients with multiple third window disorders, highlighting the importance of an extensive preoperative clinical, instrumental and radiological assessment [13].

## 4. Conclusions

As already reported in SSCD, the function of the dehiscent SC, measured by the vHIT, might be impaired in PSCD. Though it has been speculated that it likely results from a canal occlusion due to a progressive herniation of the surrounding structures through the dehiscence, the anatomical disposition of the PSC with respect to both the PCF and HJB, together with the detection of symptoms and signs consistent with a TMWM, makes this pathomechanism less likely. VOR gain reduction for the affected canal in PSCD might be due to other mechanisms, including a dissipation of endolymphatic flows at the dehiscence due to an indentation of the membranous canal during head impulses, as already assumed for SSCD. This putative mechanism is further strengthened by the detection of a VOR gain reduction for the HSC in two cases with a concurrent HSC-FN dehiscence. SC impairment should be considered as an additional sign of a TMWM and SCD should be sought in patients with third-window symptoms and signs exhibiting selective canal impairment.

## Figures and Tables

**Table 1:** Demographics and clinical/instrumental data of each patient with PSCD.

n	Age (y), Sex	PSCD Side	Other Findings	Auditory Symptoms and Signs						Vestibular Symptoms and Signs					
				HL	Pulsatile Tinnitus	Hyperacusis	Autophony	Aural Fullness	Audiometry	Dizziness	Acute Vertigo	H/T	VEMPs	Caloric Test	VOR Reduction on vHIT
1	18, F	R	−	−	+	+	+	+	pseudo-CHL	−	−	−	enhanced	−	R PSC
2	44, F	L	−	+	+	−	−	+	up-sloping mixed HL	+	L PSC BPPV	−	enhanced	normal	L PSC
3	59, M	R	L HSCD with FN	−	+	−	+	−	normal	+	−	−	enhanced	−	R PSC, L HSC
4	68, M	L	L HSCD with FN	+	+	−	+	−	down-sloping mixed HL	−	+	−	enhanced	−	L PSC, L HSC
5	13, F	R	-	-	+	-	-	-	CHL	-	+	+	enhanced	-	R PSC
6	65, F	R	-	+	-	-	-	+	mixed HL	+	-	+	enhanced	-	R PSC

## Data Availability

All data are presented in the article.

## Abbreviations

ABG: air–bone gap, AC: air-conducted, BC: bone-conducted, BCT: bithermal caloric test, BPPV: benign paroxysmal positional vertigo, CHL: conductive hearing loss, F: female, FN: facial nerve, H: Hennebert’s sign, HJB: high-riding jugular bulb, HL: hearing loss, HRCT: high-resolution computed tomography, HSC: horizontal semicircular canal, HSCD: horizontal semicircular canal dehiscence, L: left, LA: left anterior, LL: left lateral, LP: left posterior, M: male, MD: Meniere’s disease, MRI: magnetic resonance imaging, PCF: posterior cranial fossa, PSC: posterior semicircular canal, PSCD: posterior semicircular canal dehiscence, R: right, RA: right anterior, RL: right lateral, RP: right posterior, RW: round window, SC: semicircular canal, SCD: semicircular canal dehiscence, SSC: superior semicircular canal, SSCD: superior semicircular canal dehiscence, SNHL: sensorineural hearing loss, T: Tullio phenomenon, TM: tympanic membrane, TMWM: third mobile window mechanism, VEMPs: vestibular-evoked myogenic potentials, vHIT: video-head impulse test. VNG: video-nystagmography. VOR: vestibulo-ocular reflex.
